# Associative factors for atopic dermatitis and other atopic diseases in middle‐aged adults: A population‐based birth cohort study among 5373 subjects

**DOI:** 10.1002/hsr2.1015

**Published:** 2022-12-24

**Authors:** Anna K. Haarala, Suvi‐Päivikki Sinikumpu, Jari Jokelainen, Juha Pekkanen, Laura Huilaja

**Affiliations:** ^1^ The Department of Dermatology, University Hospital of Oulu PEDEGO Research Unit Oulu Finland; ^2^ Medical Research Center, PEDEGO Research Group University of Oulu Oulu Finland; ^3^ Infrastructure for Population Studies, Faculty of Medicine University of Oulu Oulu Finland; ^4^ Department of Public Health University of Helsinki Helsinki Finland; ^5^ Finnish Institute for Health and Welfare Helsinki Finland; ^6^ Department of Health Security, Environmental Health Finnish Institute for Health and Welfare Kuopio Finland

**Keywords:** adult, allergic conjunctivitis, allergic rhinitis, asthma, atopic dermatitis, sensitization

## Abstract

**Background and aims:**

The study aimed to examine parental, longitudinal and current associative factors for atopic dermatitis (AD) and to compare those to other atopic diseases in 46‐year‐old adults.

**Methods:**

Questionnaire data from the Northern Finland Birth Cohort 1966 study were used. To analyze allergic sensitization, skin prick tests (*n* = 5373) were performed for birch, timothy, cat, and house dust mite at age 46.

**Results:**

Maternal (odds ratio [OR] 1.81; 95% confidence interval [CI] 1.25–2.59) and paternal allergy (OR 2.54; CI 1.76–3.64), sensitization to any of the four tested aeroallergens (OR 1.56; CI 1.04–2.30) as well as polysensitization (OR 3.04; CI 2.10–4.37) were associated with current AD. Living on a farm in infancy was negatively associated with allergic rhinitis, allergic conjunctivitis, and atopic multimorbidity. Current AD (OR 2.65; CI 1.44–4.60) and all atopic diseases associated with indoor air related symptoms. Current AD associated with other atopic diseases, most strongly with allergic rhinitis (OR 4.92; CI 3.92–6.22).

**Conclusion:**

Current AD in a 46‐year‐old general population occurred frequently with allergic rhinitis, allergic conjunctivitis, and asthma in the Northern Finland Birth Cohort study 1966. Parental allergy and sensitization to common aeroallergens were found as shared associative factors for AD, allergic rhinitis, allergic conjunctivitis, and asthma. AD and other atopic diseases associated with symptoms related to poor indoor air quality. In daily practice, it is important to take these comorbidities into consideration when treating patients with AD.

## 1 INTRODUCTION

Atopic dermatitis (AD), allergic rhinitis (AR), allergic conjunctivitis (AC), and asthma are common in adults^1^, ^2^ with a significant effect on the quality of life^3^, ^4^, ^5^ and work performance.^3^, ^4^, ^5^ The association between asthma and AR with AD in adults is well established, but the association of AC with AD is still uncertain.^6^ Although it has been suggested that other atopic diseases develop as a consequence of the epithelial barrier disruption and inflammation in AD, that is, the atopic march,^7^ longitudinal studies have found that this is not usually the sequence.^8^, ^9^

Both genetic and environmental factors contribute to the risk of atopic diseases. Parental atopic diseases have shown to increase the risk of atopic morbidity in the offspring.^1^, ^2^, ^10^ Several environmental factors that could predispose to atopic diseases in adulthood have been studied and some differences in risk factors between atopic diseases have been found.^10^ Correspondingly, atopic sensitization is variably associated with clinical manifestations of atopic diseases^1^, ^2^, ^10^, ^11^ and especially polysensitization has been linked to atopic multimorbidity (two or more atopic diseases).^11^, ^12^

A few studies have investigated the risk factors in AD alongside AR, AC and asthma in adults, and the findings vary between studies.^2^, ^10^, ^13^ The aim in the present study was to investigate the associative factors for AD and to compare them to those of other atopic diseases (AR, AC, and asthma) in an unselected 46‐year‐old population, which is part of the Northern Finland Birth Cohort 1966. In addition, we aimed to examine the atopic comorbidity in AD in middle‐aged adults.

## 2 MATERIALS AND METHODS

### 2.1 Study population

Our study used data from the Northern Finland Birth Cohort 1966 (NFBC 1966), which is a longitudinal research program in the two northernmost provinces in Finland. Initially, all 12,058 children whose expected date of birth fell in the year 1966 were included in the NFBC 1966. Health questionnaires and clinical examinations have been conducted regularly on the whole cohort since birth.^14^

### 2.2 Skin prick test (SPT), questionnaire, and confounding factors

SPT were performed at age 46 and the procedure has been described in detail in a previous article.^15^ Data on atopic diseases (AD, AR, AC, and asthma) and medications for AD and asthma were collected by way of a health questionnaire at age 46. “Current AD” was defined as symptoms of AD (“Have you had a rash that has been called AD, milk crust, or dermatitis of the skin folds?”) in the previous 12 months or doctor‐diagnosed AD and topical corticosteroids or calcineurin inhibitors as current medication. “Lifetime AD” was defined as symptoms of AD in the previous 12 months or earlier or doctor‐diagnosed AD and topical corticosteroids or calcineurin inhibitors as current medication. The definition of “asthma” included symptoms of asthma (“Have you had symptoms of asthma?”) in the previous 12 months or doctor‐diagnosed asthma or if inhaled corticosteroids and/or bronchodilators were reported as current medication. Symptoms of AR (“Have you had allergic rhinitis related to animals or pollens or hay fever?”) and AC (“Have you had allergic eye symptoms, such as itch or watering eyes in contact with animals or during the pollen season?”) in the previous 12 months or doctor‐made diagnosis of these diseases were defined as “AR” and “C.” “Multimorbidity” was defined as two or more atopic diseases.

Data on factors (living on a farm in infancy,^10^ current residence [divided into three categories: inner urban, outer urban and rural area]^2^ or current occupation as farming,^16^ current pets [furry animals or birds],^17^ sex,^2^ current education [divided into three categories and representing socioeconomic status],^18^ current BMI,^19^ former smoking^20^ [not in the previous 12 months but before] and current smoking [in the previous 12 months],^20^ symptoms related to indoor air [at home or at workplace],^21^, ^22^ parental asthma and parental type I allergy^23^ and gestational age^24^) that could have an impact on atopic manifestations according to literature were collected by way of health questionnaires antenatally and at age 46.

### 2.3 Statistical analysis

Means and standard deviations (SD) or proportions were calculated and presented for each variable. The statistical significance of the mean differences and proportions of categorical variables between the atopic disease groups were estimated by a Mann–Whitney U‐test and a *χ* ^2^ test, respectively. Crude odds ratios (ORs) with 95% confidence intervals (CI) for atopic diseases were assessed using a binary logistic regression model. Adjusted ORs were calculated using a binary logistic regression model, and all above‐mentioned potential confounding factors were entered in the model as potential predictors for atopic diseases. All significance tests were two‐tailed, and values of *p* < 0.05 were considered statistically significant. The statistical analyses were conducted using the R software package version 4.1.2 (https://cran.rstudio.com).

## 3 RESULTS

### 3.1 Characteristics of the study population

At 46 years, invitations and questionnaires were sent to every living member of the cohort whose address was known (*n* = 10,321); 5861 participants attended the clinical examination. SPTs were performed in 5714 (55.4% of invited) participants. Because of invalid data, 331 participants were excluded from the analysis, and 10 participants did not give their consent to data processing. Complete SPT data on all allergens were available for 5373 participants (2394 [44.6%] were men) who comprised the final study population.^15^ Due to incompletely filled questionnaires, the study population varies by question. The characteristics of the study population stratified by sex are shown in Table 1.

**Table 1:** Characteristics of the study population stratified by sex

	All *n* = 5373	Men *n* = 2394	Women *n* = 2979	*p* Value*
	*n* (%)	*n* (%)	*n* (%)	
*Atopic diseases*				
Current^a^ AD	364 (7.3)	125 (5.7)	239 (8.5)	**<0.001**
Lifetime AD	1285 (25.4)	466 (20.9)	819 (28.8)	**<0.001**
Asthma	465 (9.2)	173 (7.8)	292 (10.3)	**0.002**
Allergic rhinitis	1661 (32.8)	644 (28.9)	1017 (35.9)	**<0.001**
Allergic conjunctivitis	1422 (28.1)	498 (22.4)	924 (32.5)	**<0.001**
Multimorbidity^b^	1340 (24.9)	484 (20.2)	856 (28.7)	**<0.001**
*Current residence*				**0.023**
Inner urban area	998 (19.7)	401 (18.0)	597 (21.0)	
Outer urban area	2610 (51.5)	1166 (52.3)	1444 (50.9)	
Rural area	1459 (28.8)	662 (29.7)	797 (28.1)	
*Current farming*	189 (3.7)	99 (4.5)	90 (3.2)	**0.019**
*Living on a farm in infancy*	1370 (26.7)	613 (26.8)	757 (26.6)	0.910
*Education*				**<0.001**
Basic	344 (6.7)	189 (8.4)	155 (5.4)	
Secondary	3343 (65.4)	1519 (67.7)	1824 (63.7)	
Tertiary	1422 (27.8)	536 (23.9)	886 (30.9)	
*Smoking*				**<0.001**
No	2738 (53.9)	1074 (48.1)	1664 (58.5)	
Former	1310 (25.8)	642 (28.8)	668 (23.5)	
Current^c^	1031 (20.3)	517 (23.2)	514 (18.1)	
*Paternal allergy*	540 (15.9)	178 (12.1)	362 (18.8)	**<0.001**
*Maternal allergy*	838 (23.5)	241 (16.0)	597 (28.9)	**<0.001**
*Paternal asthma*	415 (11.7)	155 (10.0)	260 (13.0)	**0.006**
*Maternal asthma*	579 (15.7)	214 (13.4)	365 (17.4)	**0.001**
*Symptoms related to indoor air*	189 (3.5)	54 (2.3)	135 (4.5)	**<0.001**
*Sensitization in SPT*				**<0.001**
No	3724 (69.3)	1600 (66.8)	2124 (71.3)	
One sensitization	847 (15.8)	400 (16.7)	447 (15.0)	
Two or more sensitizations	802 (14.9)	394 (16.5)	408 (13.7)	
*Having pets*	2596 (48.3)	1082 (45.2)	1514 (50.8)	**<0.001**

Abbreviations: AD, atopic dermatitis; SPT, skin prick test.

a AD in the previous 12 months or doctor‐diagnosed AD and topical corticosteroids or calcineurin inhibitors as current medication.

^b^ Multimorbidity is defined as two or more atopic diseases.

^c^ Smoking in the previous 12 months.

* Difference between genders is tested using *χ* ^2^ test.

### 3.2 Prevalence of atopic diseases and atopic multimorbidity at age 46

Any current atopic disease was reported by 2101 participants (39.1%). Current AD, lifetime AD, AR, AC, asthma, and multimorbidity occurred in 7.3%, 25.4%, 32.8%, 28.1%, 9.2%, and 24.9%, respectively. Women reported significantly more atopic diseases than men (Table 1).

### 3.3 Associative factors for atopic diseases

#### 3.3.1 Atopic predisposition

**Figure 1:**
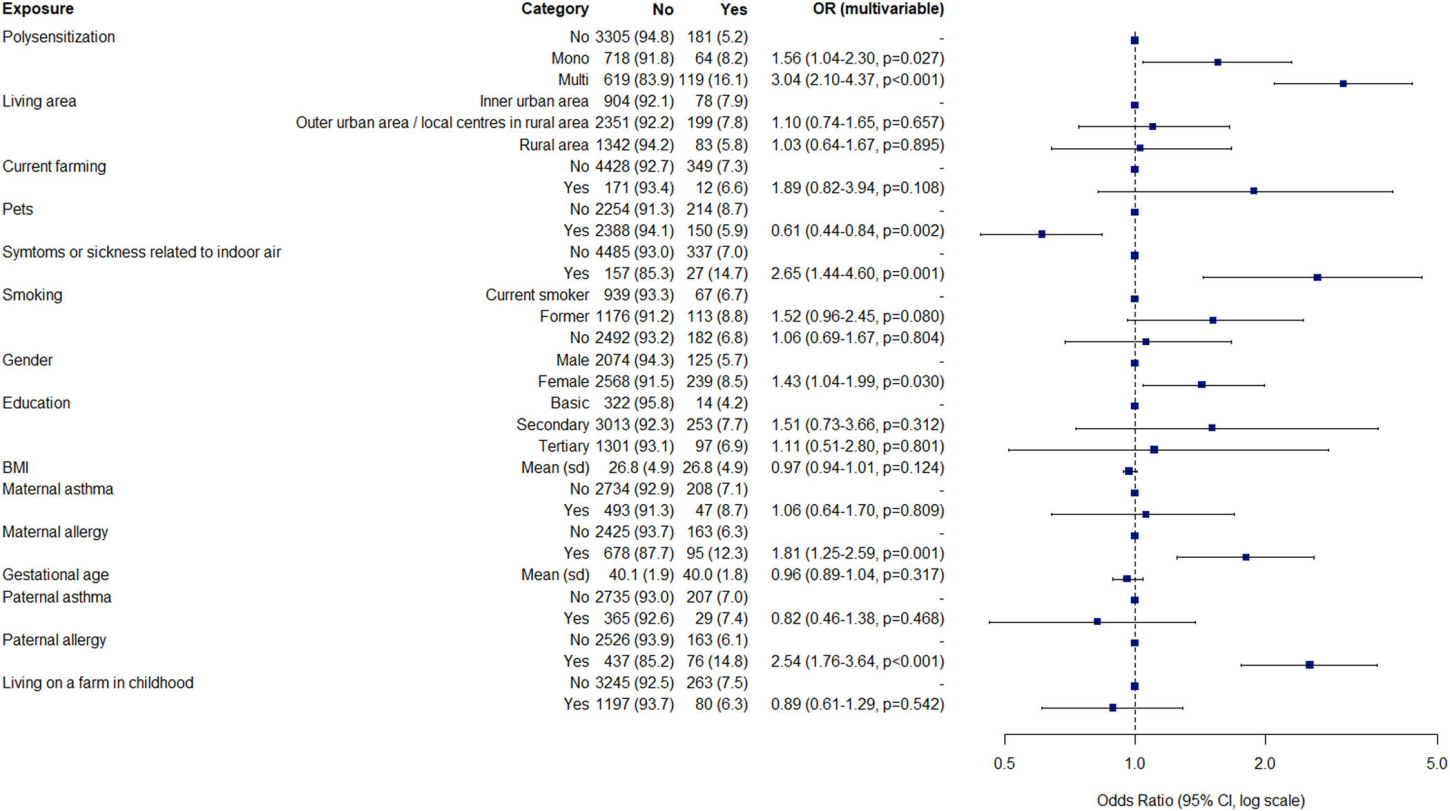
Determinants of current atopic dermatitis in the Northern Finland Birth Cohort 1966 Study. Multivariate logistic regression analysis. Risks are presented as adjusted odds ratios with 95% confidence interval. BMI, body mass index; p, *p* value; Sd, standard deviation.

**Figure 2:**
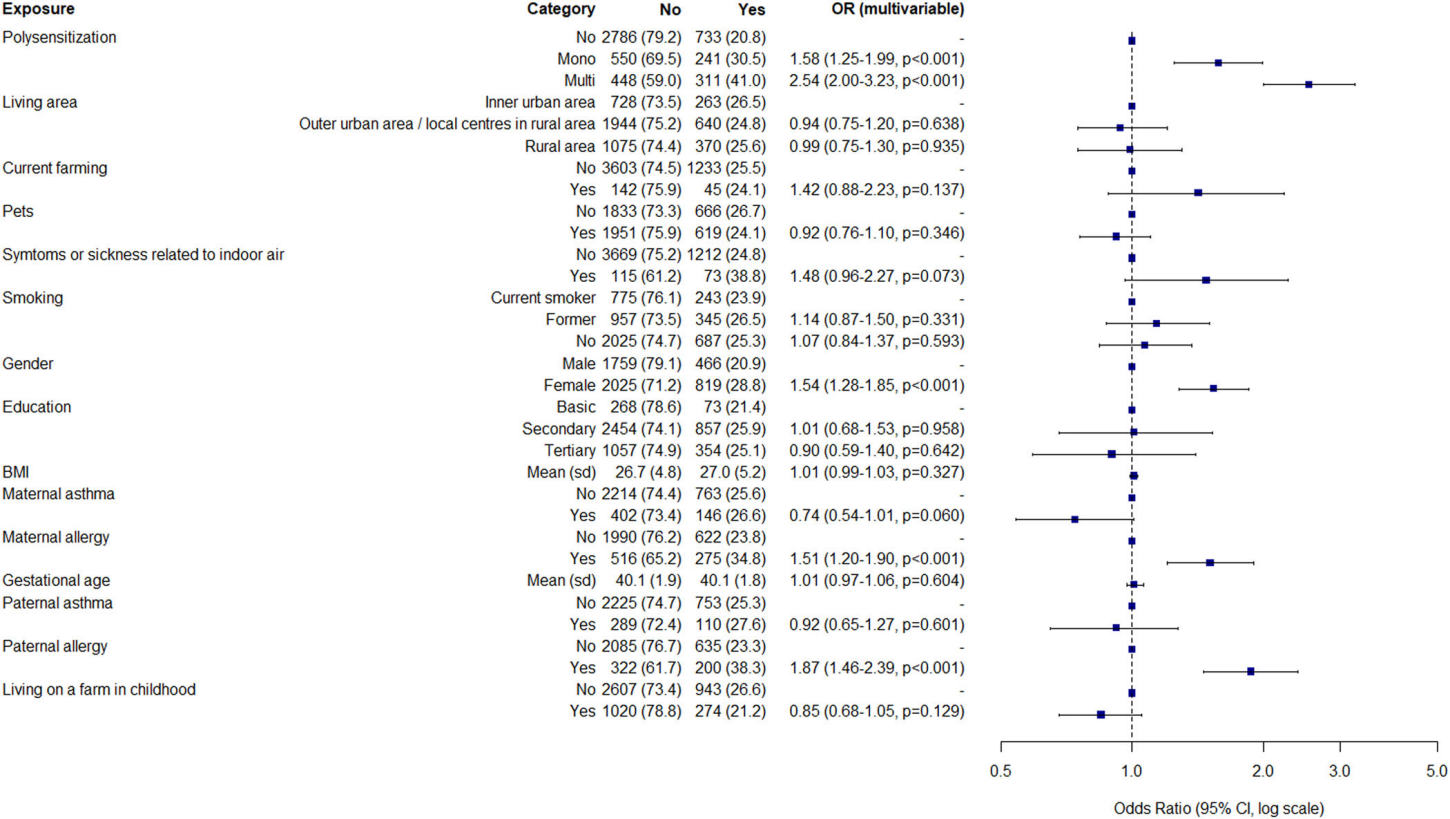
Determinants of lifetime atopic dermatitis in the Northern Finland Birth Cohort 1966 Study. Multivariate logistic regression analysis. Risks are presented as adjusted odds ratios with 95% confidence interval. BMI, body mass index; p, *p* value; Sd, standard deviation.

Allergy of either parent associated with AD (both lifetime and current) (Figures 1 and 2), AR, AC, and multimorbidity (Supporting Information: Figures 1, 2, and 4), whereas only maternal allergy associated with asthma (Supporting Information: Figure 3). Paternal asthma was significantly associated with asthma (OR 1.61; CI 1.02–2.46) (Supporting Information: Figure 3).

#### 3.3.2 Gender

In the multivariate model, female gender was associated with current AD (OR 1.43; CI 1.04–1.99), lifetime AD (OR 1.54; CI 1.28–1.85) (Figures 1 and 2), AR, AC, multimorbidity but not for asthma. (Supporting Information: Figures 1–4).

#### 3.3.3 Residence, farming, and indoor air

Living on a farm in infancy was not associated with current AD, lifetime AD (Figures 1 and 2), or asthma (Supporting Information: Figure 3), however it was negatively associated with AR (OR 0.68; CI 0.54–0.84), AC (OR 0.62; CI 0.49–0.79), and multimorbidity (OR 0.59; CI 0.46–0.75) (Supporting Information: Figures 1, 2, and 4). Current residence in rural area and farming were not associated with atopic diseases. Those who reported symptoms related to indoor air had an association for current AD (OR 2.65; CI 1.44–4.6) (Figure 1), AR, AC, asthma and atopic multimorbidity (Supporting Information: Figures 1–4).

#### 3.3.4 Smoking, pets, BMI, and education

Smoking was not associated with atopic diseases. Having pets at age 46 negatively associated with current AD (Figure 1), AC and atopic multimorbidity (Supporting Information: Figures 2 and 4), but not with asthma or AR. BMI was not associated with AD, but higher BMI was slightly associated with asthma (OR 1.03; CI 1.01–1.06) (Supporting Information: Figure 3). In turn, higher level of education was a protective factor for asthma (OR 0.53; CI 0.30–0.95) (Supporting Information: Figure 3) but was not associated with other atopic diseases.

#### 3.3.5 Sensitization in atopic diseases

Monosensitization at age 46 was associated with current AD (OR 1.56; CI 1.04–2.30) (Figure 1); however, the association for other atopic disease was higher: AR (OR 3.63; CI 2.91–4.54), AC (OR 3.68; CI 2.91–4.64), and asthma (OR 1.89; CI 1.33–2.64) (Supporting Information: Figures 1–3). For current AD, polysensitization was associated with it more than three‐fold (OR 3.04; CI 2.10–4.37) (Figure 1). Of the single aeroallergens, the association with cat was the most strongest with current AD (OR 3.06; CI 2.42–3.86) (Supporting Information: Table SI).

### 3.4 Atopic comorbidity in AD

Current AD associated strongly with AR (OR 4.92; CI 3.92–6.22), AC (OR 4.82; CI 3.85–6.04) and asthma (OR 3.02; CI 2.28–3.96). (Table 2) In participants with current AD (*n* = 355), another atopic disease was reported by 272 participants (76.6%). The most common combination was AD with AR and AC (*n* = 130/36.6%). Sixty‐four (18%) participants had all four atopic diseases (Supporting Information: Table SII).

**Table 2:** Association of AD with atopic comorbidities

	Asthma^a^			Allergic rhinitis^a^			Allergic conjunctivitis^a^		
	No *n* (%)	Yes *n* (%)	OR (95% CI)	No *n* (%)	Yes *n* (%)	OR (95% CI)	No *n* (%)	Yes *n* (%)	OR (95% CI)
Current AD	284 (78.9)	76 (21.1)	3.02 (2.28–3.96)	116 (32.2)	244 (67.8)	4.92 (3.92–6.22)	137 (37.8)	225 (62.2)	4.82 (3.85–6.04)

*Note*: Multivariate logistic regression analysis, adjusted for sex and education. Risks are presented as odds ratios (OR) with 95% confidence interval (CI).

Abbreviation: AD, atopic dermatitis.

^a^ Combinations of comorbidities are included in the analysis.

## 4 DISCUSSION

In this large birth cohort study, we studied current and longitudinal factors associated with atopic diseases, especially AD. Atopic predisposition and sensitization at age 46 were associative factors for all the atopic diseases. Farm environment in childhood was negatively associated with AR, AC, and multimorbidity, but not of AD or asthma. Self‐reported symptoms related to indoor air were associated with all the atopic diseases including AD. Smoking was not associated with atopic diseases.

Atopic predisposition, that is, allergy of parents, was significantly associated with AD, and this effect could also be seen in AR, AC, asthma, and atopic multimorbid phenotype, which is in line with previous studies.^1^, ^2^, ^13^ Parental asthma has been a risk factor for childhood asthma in previous studies,^25^ and in the present study, paternal asthma significantly associated with asthma as well.

Difference between genders in the prevalence of atopic diseases was seen as women reported significantly more often all the atopic diseases and multimorbidity. However, in the multivariate analysis, female gender associated with current and lifetime AD, AR, AC, multimorbidity, but not for asthma. In previous studies, female gender has been a risk factor for eczema,^2^, ^26^ AD,^27^ and AR.^28^ In asthma, the risk is greater in young boys than girls, but after puberty the risk becomes greater in women.^29^ However, a recent study found that this shift might occur again during the menopause as the age‐related risk of severe asthma was higher in men than in women after the age of 45.^30^

Aeroallergen sensitization and polysensitization associated with AD in the current study. In previous studies, the association between sensitization and AD in adults has varied.^2^, ^10^, ^13^, ^31^ A Swedish study (*n* = 18,087) found that any positive SPT to aeroallergens increased the risk of any eczema,^2^ whereas in another Swedish study (*n* = 1172), atopic sensitization was not a risk factor for current eczema.^10^ Raciborski and coworkers (*n* = 1805 adults) did not find an association between sensitization or polysensitization and AD as a single disease in their study in Poland.^31^ In a Chinese study in adults (*n* = 2096), sensitization to any of the 9 pollen and HDM allergens was associated with both eczema and “eczema with asthma and/or hay fever.”^13^ In the current study, sensitization and polysensitization associated also with AR, AC, and asthma. Corresponding findings were reported in a recent Danish study (*n* = 52,976), in which sensitization to common inhalant allergens was a risk factor for AR, AC, and asthma.^1^

Living on a farm in infancy did not protect from current or lifetime AD or asthma. However, the protective effect on AR, AC, and multimorbidity was seen in our study. A study conducted in Sweden found a protective effect of farm upbringing on lifetime eczema, but not on current eczema.^2^ However, another Swedish study with the same definitions and conducted in the same area found the protective effect also on current eczema in adults.^10^ In these studies, the age range was wider (16–75 years), and the definition of AD was different from the definition used in our study. Risk factors for AR, AC, and asthma were studied in adults; in a Danish study (*n* = 52,976), upbringing on a farm with livestock compared to other levels of urbanization had a protective effect on allergic rhino‐conjunctivitis,^1^ which was also seen in the current study. The protective effect of farm upbringing on the triad of AD, asthma, and AR or their different combinations was not confirmed in another setup.^32^ However, in our study, living on a farm in infancy clearly protected from multimorbidity.

Current residence or current farming were not associated with atopic diseases in our study. Having pets at age 46 negatively associated with AD, AC, and atopic multimorbidity, but not with asthma or AR. This may come from the fact that those who already have atopic diseases, especially the multimorbid phenotype, might not acquire pets in adulthood as readily.

Smoking was not associated with current or lifetime AD in our study. Previous studies on the relation between AD and smoking suggest a positive association: A systematic review and meta‐analysis of 86 studies with 680,176 patients (598,296 children) found that AD in both adults and children associated with active smoking and exposure to passive smoke, but not maternal smoking during pregnancy.^33^ However, differences in inclusion criteria and sample sizes in addition to regional variation were found between the included studies.^33^

In the present study, symptoms related to indoor air were significantly associated with AD, AR, AC, asthma, and atopic multimorbidity, as in previous studies^21^, ^34^: In Sweden, several indoor air factors, including humid air and mold odor in multifamily houses, associated with eczema, allergy, and asthma.^21^ Recently, a study conducted in Finland found that the toxicity of wiped dust and airborne microbes in indoor air at workplace were significantly associated with multiple work‐related respiratory and ocular symptoms.^22^ In the same study, the toxicity of wiped dust was significantly associated with AR. This association might be bidirectional as the toxicity of indoor air might predispose to the development of atopic diseases, or individuals with atopic morbidity might be more prone to develop symptoms related to indoor air toxins or microbes.

In this cohort of middle‐aged adults, 7.3% reported current AD and 25.4% reported lifetime AD. According to a cross‐sectional multicenter study, approximately 4.4% of the adults in the EU and 4.9% in the United States have AD.^27^ Corresponding results have been found in a Finnish study in which a skin examination performed by dermatologists found 4.6% prevalence of AD in adults in general population.^35^ AR and AC were common in the study cohort, with the prevalence of 32.8% and 28.1%, respectively. AR affects around 20%–30% of European adult population.^28^ AC often occurs with AR, and the prevalence in general adult population has been between 15% and 40% in studies.^36^ Current asthma occurred in 9.2% in the present study. In Northern European studies, the prevalence of asthma was 10.9% both in Sweden^37^ and in Finland^35^ in 2016. Thus, our observations on the prevalences of atopic diseases are in line with previous studies.

AD associated with all the other atopic diseases in the current study, most strongly with AR. In previous studies, AD in adults has clearly associated with hay fever and asthma,^6^, ^13^, ^38^, ^39^ whereas less evidence is available for an association with AC.^6^, ^40^ In the current study, AD occurred most often in combination with AR and AC (36.6%), whereas AD as a single disease was reported by only 23.4% of the participants with AD. Comorbid asthma occurred in 21.1% and the prevalence of concomitant AR was surprisingly high (67.6%). For comparison, in a study of US adults, 18.7% and 28.4% of AD patients had concomitant asthma and hay fever, respectively.^38^

The strengths of the study are the large and unselected study population which represents well the middle‐aged adult population, both men and women. Compared to the Danish study,^1^ which investigated atopic respiratory diseases without AD among blood donators, those with severe asthma or food allergies were excluded from the study population. The analyses in our study included comprehensively the confounders that have previously been shown to associate with atopic diseases. Selection bias might have occurred in responding to the questionnaire and participation in the examination day and SPT, which are limitations of the study. The definitions of current AD or current eczema vary between studies, and this must be considered when comparing the results. The chosen questions combined with the medication data in the current study were aimed to specifically include only AD and to exclude other types of eczemas that could exaggerate the true prevalence of lifetime and current AD.

In conclusion, AD had shared associative factors with AR, AC, and asthma, including parental allergy and sensitization to aeroallergens. In addition, a common association was found with indoor air related symptoms while current residence or farming were not associated with any of the atopic diseases. Women reported atopic morbidity more often than men. Differences between associative factors were also found as living on a farm in infancy did not protect from AD or asthma, but was negatively associated with AR, AC, and multimorbidity. Patients with AD commonly had atopic comorbidities and suffered from poor indoor air, which should be remembered as factors that could contribute to the exacerbation of AD when treating AD patients.

## AUTHOR CONTRIBUTIONS

**Anna K. Haarala**: Conceptualization; visualization; writing – original draft. **Suvi‐Päivikki Sinikumpu**: Conceptualization; investigation; project administration; supervision; writing – review & editing. **Jari Jokelainen**: Data curation; formal analysis; methodology; software; visualization. **Juha Pekkanen**: Conceptualization; resources; supervision. **Laura Huilaja**: Conceptualization; investigation; project administration; supervision; visualization; writing – review & editing. All authors have read and approved the final version of the manuscript.

## CONFLICT OF INTEREST

The authors declare no conflict of interest.

## ETHICS STATEMENT

The Ethical Committee of the Northern Ostrobothnia Hospital District approved the study (§94/2011), which was performed according to the Helsinki Declaration of 1983. Written informed consent for scientific purposes was received from all participants.

## TRANSPARENCY STATEMENT

The lead author Suvi‐Päivikki Sinikumpu affirms that this manuscript is an honest, accurate, and transparent account of the study being reported; that no important aspects of the study have been omitted; and that any discrepancies from the study as planned (and, if relevant, registered) have been explained.

## Supporting information

Supporting information.

## Data Availability

NFBC data is available from the University of Oulu, Infrastructure for Population Studies. Permission to use the data can be applied for research purposes via electronic material request portal. In the use of data, the EU general data protection regulation (679/2016) and Finnish Data Protection Act are followed. The use of personal data is based on the cohort participant's written informed consent at his/her latest follow‐up study, which may cause limitations to its use. Suvi‐Päivikki Sinikumpu had full access to all of the data in this study and takes complete responsibility for the integrity of the data and the accuracy of the data analysis.
