# Supplementary material for: Associative factors for atopic dermatitis and other atopic diseases in middle‐aged adults: A population‐based birth cohort study among 5373 subjects
Source: Health Sci Rep. 2022 Dec 24;6(1):e1015. doi: 10.1002/hsr2.1015 (PMC9789389; doi:10.1002/hsr2.1015)
Supplement: Supplementary file 1 — Supporting information. [file HSR2-6-e1015-s001.docx]

**Table SI. Association of current sensitization to aeroallergens with current atopic diseases at age 46.**

|  | **Atopic dermatitis** | | | | **Asthma** | | | | **Allergic rhinitis** | | | | **Allergic conjunctivitis** | | | | **Multimorbidity**^†^ | | |
| --- | --- | --- | --- | --- | --- | --- | --- | --- | --- | --- | --- | --- | --- | --- | --- | --- | --- | --- | --- |
|  | *No* | *Yes* | *OR [CI]* | *No* | | *Yes* | *OR [CI]* | *No* | | *Yes* | *OR [CI]* | *No* | | *Yes* | *OR [CI]* | *No* | | *Yes* | *OR [CI]* |
|  | *N=4642* | *N=364* |  | *N=4580* | | *N=465* |  | *N=3402* | | *N=1661* |  | *N=3636* | | *N=1422* |  | *N=4966* | | *N=407* |  |
| **Sensitization** |  |  |  |  | |  |  |  | |  |  |  | |  |  |  | |  |  |
| 0 | 3305 (94.8%) | 181 (5.2%) | Ref. | 3282 (93.6%) | | 225  (6.4%) | Ref. | 2788 (79.5%) | | 721 (20.5%) | Ref. | 2933 (83.7%) | | 573 (16.3%) | Ref. | 3587 (96.3%) | | 137  (3.7%) | Ref. |
| 1 | 718 (91.8%) | 64  (8.2%) | 1.63 [1.20;2.18] | 697 (88.5%) | | 91  (11.5%) | 1.91 [1.47;2.46] | 401 (50.6%) | | 391 (49.4%) | 3.77 [3.21;4.43] | 456 (57.7%) | | 334 (42.3%) | 3.75 [3.17;4.43] | 768 (90.7%) | | 79  (9.3%) | 2.69 [2.01;3.58] |
| ≥2 | 619 (83.9%) | 119 (16.1%) | 3.51 [2.74;4.49] | 601 (80.1%) | | 149 (19.9%) | 3.62 [2.88;4.52] | 213 (28.0%) | | 549 (72.0%) | 9.96 [8.34;11.9] | 247 (32.4%) | | 515 (67.6%) | 10.7 [8.95;12.7] | 611 (76.2%) | | 191 (23.8%) | 8.18 [6.47;10.4] |
| **Cat** |  |  |  |  | |  |  |  | |  |  |  | |  |  |  | |  |  |
| No | 3999 (94.2%) | 244 (5.8%) | Ref. | 3976 (93.1%) | | 293  (6.9%) | Ref. | 3122 (73.0%) | | 1154 (27.0%) | Ref. | 3329 (78.0%) | | 939 (22.0%) | Ref. | 4334 (95.4%) | | 210  (4.6%) | Ref. |
| Yes | 643 (84.3%) | 120 (15.7%) | 3.06 [2.42;3.86] | 604 (77.8%) | | 172 (22.2%) | 3.86 [3.14;4.75] | 280 (35.6%) | | 507 (64.4%) | 4.90 [4.17;5.76] | 307 (38.9%) | | 483 (61.1%) | 5.57 [4.75;6.55] | 632 (76.2%) | | 197 (23.8%) | 6.43 [5.20;7.95] |
| **Birch** |  |  |  |  | |  |  |  | |  |  |  | |  |  |  | |  |  |
| No | 3907 (93.9%) | 256 (6.2%) | Ref. | 3871 (92.3%) | | 322  (7.7%) | Ref. | 3129 (74.4%) | | 1074 (25.6%) | Ref. | 3316 (79.0%) | | 883 (21.0%) | Ref. | 4233 (94.8%) | | 233  (5.2%) | Ref. |
| Yes | 735 (87.2%) | 108 (12.8%) | 2.24 [1.76;2.84] | 709 (83.2%) | | 143 (16.8%) | 2.43 [1.96;2.99] | 273 (31.7%) | | 587 (68.3%) | 6.26 [5.34;7.35] | 320 (37.3%) | | 539 (62.7%) | 6.32 [5.41;7.40] | 733 (80.8%) | | 174 (19.2%) | 4.31 [3.49;5.33] |
| **Timothy** |  |  |  |  | |  |  |  | |  |  |  | |  |  |  | |  |  |
| No | 3959 (93.7%) | 265 (6.3%) | Ref. | 3910 (92.0%) | | 340  (8.0%) | Ref. | 3157 (74.1%) | | 1101 (25.9%) | Ref. | 3342 (78.6%) | | 911 (21.4%) | Ref. | 4276 (94.6%) | | 245  (5.4%) | Ref. |
| Yes | 683 (87.3%) | 99 (12.7%) | 2.17 [1.69;2.76] | 670 (84.3%) | | 125 (15.7%) | 2.15 [1.72;2.67] | 245 (30.4%) | | 560 (69.6%) | 6.55 [5.56;7.74] | 294 (36.5%) | | 511 (63.5%) | 6.37 [5.43;7.49] | 690 (81.0%) | | 162 (19.0%) | 4.10 [3.30;5.07] |
| **HDM** |  |  |  |  | |  |  |  | |  |  |  | |  |  |  | |  |  |
| No | 4448 (93.1%) | 332 (7.0%) | Ref. | 4386 (91.2%) | | 423  (8.8%) | Ref. | 3289 (68.2%) | | 1536 (31.8%) | Ref. | 3511 (72.8%) | | 1314 (27.2%) | Ref. | 4751 (92.9%) | | 365  (7.1%) | Ref. |
| Yes | 194 (85.8%) | 32 (14.2%) | 2.22 [1.48;3.23] | 194 (82.2%) | | 42  (17.8%) | 2.25 [1.57;3.16] | 113 (47.5%) | | 125 (52.5%) | 2.37 [1.82;3.08] | 125 (53.6%) | | 108 (46.4%) | 2.31 [1.77;3.01] | 215 (83.7%) | | 42  (16.3%) | 2.55 [1.78;3.57] |

*Notes:* Risks are presented as odds ratios (OR) with 95% confidence interval (CI). ^†^Multimorbidity is defined as two or more atopic diseases.

*Abbreviations*: HDM, house dust mite; Ref, reference

**Table SII. Atopic comorbidities in participants with current atopic dermatitis.**

| *The combinations of comorbidities in AD* | *Total*  *n=355^#^*  *n (%)* | *Men*  *n=123*  *n (%)* | *Women*  *n=232*  *n (%)* |
| --- | --- | --- | --- |
| *AD ONLY* | 83 (23.4) | 38 (30.9) | 45 (19.4) |
| *AD, AR ONLY* | 41 (11.5) | 21 (17.1) | 20 (8.6) |
| *AD, AC ONLY* | 26 (7.3) | 7 (5.7) | 19 (8.2) |
| *AD, A ONLY* | 6 (1.7) | 2 (1.6) | 4 (1.7) |
| *AD, AR, AC* | 130 (36.6) | 32 (26.0) | 98 (42.2) |
| *AD, A, AR* | 5 (1.4) | 2 (1.6) | 3 (1.3) |
| *AD, A, AR, AC* | 64 (18.0) | 21 (17.1) | 43 (18.5) |
| *The prevalence of comorbidities in AD* | *n (%)* | *n (%)* | *n (%)* |
| *AR* | 240 (67.6) | 76 (61.8) | 164 (70.7) |
| *AC* | 220 (62.0) | 60 (48.8) | 160 (69.0) |
| *A* | 75 (21.1) | 25 (20.3) | 50 (21.6) |

*Notes:* ^#^Included participants with AD and data on all atopic diseases.

*Abbreviations*: AD, atopic dermatitis; AR, allergic rhinitis, AC,

allergic conjunctivitis, A, asthma

**Supplemental Figure 1. Determinants of allergic rhinitis.** *Notes:* Multivariate logistic regression analysis. Risks are presented as adjusted odds ratios (OR) with 95% confidence interval (CI). *Abbreviations*: Sd, standard deviation; p, p-value; BMI: body mass index

**Supplemental figure 2. Determinants of allergic conjunctivitis.**

*Notes:* Multivariate logistic regression analysis. Risks are presented as odds ratios (OR) with 95% confidence interval (CI).

*Abbreviations*: Sd, standard deviation; p, p-value; BMI: body mass index

**Supplemental figure 3. Determinants of asthma.**

*Notes:* Multivariate logistic regression analysis. Risks are presented as odds ratios (OR) with 95% confidence interval (CI).

*Abbreviations*: Sd, standard deviation; p, p-value; BMI: body mass index

**Supplemental figure 4. Determinants of atopic multimorbidity.**

*Notes:* Multivariate logistic regression analysis. Risks are presented as odds ratios (OR) with 95% confidence interval (CI).

*Abbreviations*: Sd, standard deviation; p, p-value; BMI, body mass index
